# Prolonged Fetal Bradycardia as the Presenting Clinical Sign in Congenital Syphilis Complicated by Necrotizing Funisitis: A Case Report

**DOI:** 10.5402/2011/320246

**Published:** 2011-04-06

**Authors:** Jun Kakogawa, Miyuki Sadatsuki, Norio Masuya, Hideto Gomibuchi, Shigeki Minoura, Kazuhusa Hoshimoto

**Affiliations:** ^1^Department of Obstetrics and Gynecology, National Center for Global Health and Medicine, 1-21-1, Toyama, Shinjuku-ku, Tokyo, 162-8655, Japan; ^2^Department of Pathology, National Center for Global Health and Medicine, Shinju-ku, Tokyo 162-8655, Japan

## Abstract

Syphilis remains a serious cause of neonatal morbidity and mortality worldwide. In this paper, we describe a case of congenital syphilis that was fully supported by abnormal fetal heart rate patterns and placental histopathological evidence. A 24-year-old para 4 woman, who did not attend antenatal care, was admitted to our hospital with a complaint of abdominal discomfort at an estimated 31-week gestation. Fetal heart rate monitoring showed prolonged bradycardia. A neonate weighting 1,423 g with severe birth asphyxia was immediately delivered by cesarean section. Following delivery, the mother and the neonate were diagnosed with syphilis. Histopathological examination confirmed severe chorioamnionitis and necrotizing funisitis with numerous *Treponema pallidum*. *Conclusions*. Challenges in establishing the diagnosis of necrotizing funisitis are essential for optimal management of a fetus with a systemic inflammatory response in utero.

## 1. Introduction

Maternal syphilis and congenital syphilis are public health problems worldwide. An estimated 50% of pregnancies with maternal syphilis will end in fetal or perinatal death, low birth weight babies, or babies born with congenital syphilis [1]. Necrotizing funisitis, which is associated with increased rates of stillbirth, perinatal infection, and preterm delivery, is sometimes accompanied with congenital syphilis. Immunohistochemistry (IHC) has been used to detect *Treponema pallidum* in the placenta and umbilical cord. Guarner et al. [2] reported that 11% of neonates born from maternal syphilis had necrotizing funisitis with *T. pallidum*. 

Even though fetal heart rate (FHR) monitoring is widely used to monitor the well-being of the fetus, congenital syphilis is rarely associated with an abnormal FHR before labor [3]. In this case report, we describe a 31-week gestation neonate with congenital syphilis in which fetal bradycardia was shown as a clinical sign by FHR monitoring. The neonate was delivered by emergency cesarean section and demonstrated severe chorioamnionitis (CAM) and necrotizing funisitis with numerous* T. pallidum *by histopathologic examination.

## 2. Case Report

An unmarried 24-year-old woman (gravida 4, para 4), who had not attended any antenatal care (ANC), presented to our hospital with a complaint of abdominal discomfort. In the patient's four previous births, the first and second children were delivered by full-term spontaneous deliveries at another hospital without ANC, and the third and fourth children were delivered at the patient's home without medical assistance. The mother and her children had not been diagnosed with syphilis. During the examination, the patient appeared healthy with a blood pressure of 118/81 mmHg, a pulse rate of 78 bpm, and a body temperature of 37.2°C. The gestational age was estimated by her last menstrual period to be approximately 31 weeks. Abdominal examination revealed a soft, nontender uterus. Ultrasound evaluation confirmed a single fetus in cephalic presentation with an estimated body weight of 1,500 g and no evidence of placental abruption. Vaginal examination showed the cervix to be 30% effaced and 2 cm dilated with an intact membrane. Laboratory findings revealed elevated white blood cell counts (11,670/*μ*L) and C-reactive protein levels (1.63 mg/dL).

FHR monitoring at the initial evaluation showed several early decelerations and bradycardia to 50 bpm for a one-minute period prior to recovery to normal baseline in association with a painless uterine contraction. The patient was immediately placed in the lateral position followed by the administration of oxygen via a face mask and a rapid intravenous infusion of fluid. The baseline variability was within a normal range. Twenty-five minutes later, another prolonged bradycardia to 70 bpm for a 7-minute period prior to recovery to normal baseline occurred without a uterine contraction. A decision was made to deliver immediately by cesarean section. Tocolysis was not performed during the course of treatment. A female neonate covered with a thick meconium weighing 1,423 g with Apgar scores of 2 and 8 at 1 and 5 minutes was delivered. Umbilical arterial pH was 7.28. After resuscitation, the neonate was transferred to the neonatal intensive care unit. The neonate was assessed to be 31 weeks at birth using the Dubowitz score and was diagnosed with an intrauterine infection. Routine investigations were performed to determine the nature of the infection. 

**Figure 1:**
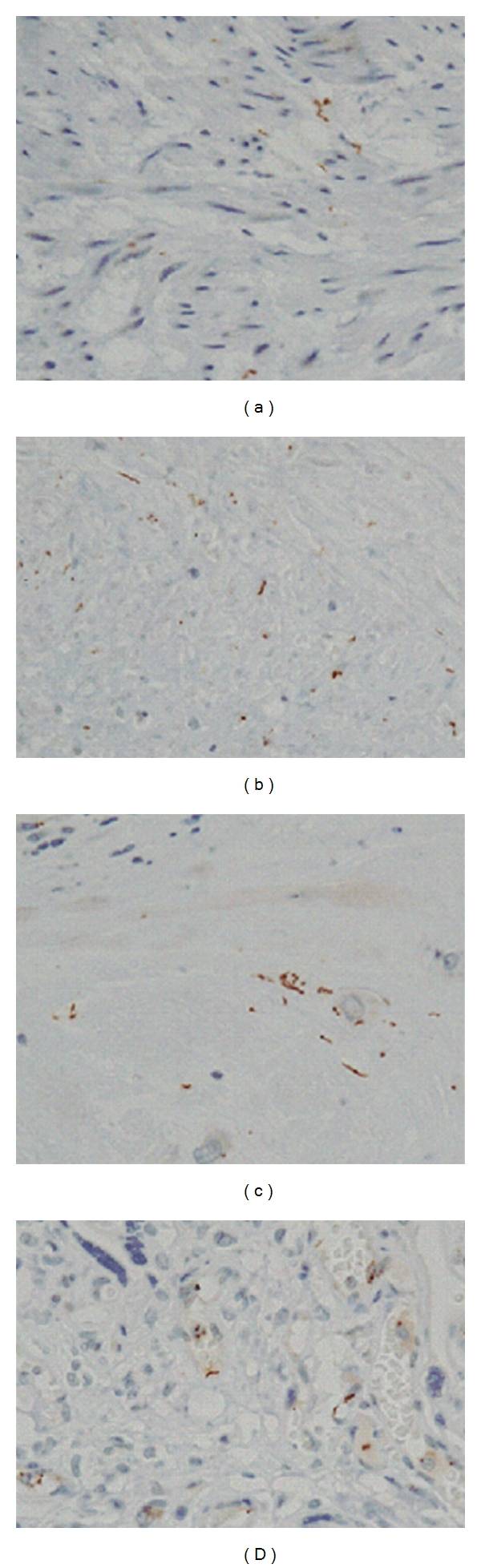
Histopathological examination of the placenta. Numerous spirochetes of *Treponema pallidum* in areas of the umbilical vein (a), chronic plate (b), deciduas (c) and villi (d) were noted by immunohistochemistry testing, which was performed using an antibody against *T. pallidum*.

Following cesarean section, the mother was diagnosed with syphilis by a serological test. Congenital syphilis was confirmed by serology of the neonate: rapid plasma region 32x, *Treponema pallidum* hemagglutination 1280x, and a positive immunoglobulin fluorescent treponemal antibody-absorption test. Histopathological examination of the placenta, umbilical cord, and membranes revealed severe CAM and necrotizing funisitis; inflammatory cells could be seen on the surface of the cord and within Wharton jelly. Numerous *T. pallidum* were detected by IHC, which uses an antibody against *T. pallidum *(Figure 1). Both mother and neonate were treated with penicillin with good results. The neonate did not have any skin lesions or radiological bone changes and was discharged after 56 days with no evidence of sequela.

## 3. Discussion

Congenital syphilis can be prevented through antenatal screening and treatment. However, more than 1 million infants are born with congenital syphilis each year worldwide. The World Health Organization aims to reduce maternal morbidity, fetal loss, and neonatal morbidity and mortality due to syphilis and is conducting a global effort to eliminate congenital syphilis [1]. It is well known that syphilis in untreated or inadequately treated pregnant women is associated with neonatal morbidity and mortality and may lead to histological effects in the placenta such as necrotizing funisitis.

To our knowledge, there has been only one case report of congenital syphilis presenting with an abnormal FHR before labor. Savage and Reader [3] reported that antepartum FHR monitoring showed a loss of baseline variability and several decelerations in a 36-week gestation neonate with congenital syphilis and severe birth asphyxia. This case study is the first report of an intrauterine syphilis infection fully supported by antepartum abnormal FHR patterns and placental histopathological evidence. Marked CAM and necrotizing funisitis with numerous *T. pallidum *were confirmed even though the symptoms and signs of inflammation of the mother were not as serious according to the laboratory test. In this case, bradycardia detected during FHR monitoring was a critical sign of fetal health and was important in the decision to perform an emergency cesarean section. Therefore, without FHR monitoring, prompt intervention, and skilled neonatal intensive care, this neonate might not have survived. This case highlights the importance of raising the level of awareness that a fetus with early onset neonatal sepsis may be born when abnormal patterns are identified during antepartum FHR monitoring. Furthermore, it is important to process all investigations urgently for women who are not attending or attending insufficient ANC. The risk of performing cesarean section on preterm infants should be considered especially when adequate facilities to manage high-risk pregnancies (e.g., NICU) are not available. 

Necrotizing funisitis, which is associated with infectious neonatal complications, occurred in 0.1% of deliveries greater than 20-week gestation [4]. It is difficult to diagnose necrotizing funisitis accurately during the antepartum period. In the current case, we could not identify suspicious findings in the cord before delivery. Thus, this case serves as a reminder of the challenges in diagnosing necrotizing funisitis and performing optimal assessment and management for a fetus with both a systemic inflammatory response and sepsis in utero. Furthermore, an abnormal FHR monitoring pattern in cases complicated by preterm birth is not a sensitive predictor of perinatal sepsis [5]. The current case emphasizes that histopathological examination of the placenta should be utilized for investigating the correlation among FHR monitoring patterns and a fetus whose mother was infected with syphilis according to the degree of intrauterine inflammation.

An association of CAM with cerebral palsy has been reported [6]. The current neonate was discharged with no evidence of sequelae despite severe CAM and necrotizing funisitis. The current case shows that congenital syphilis still occurs even though antenatal syphilis screening is performed for all pregnant women by public funds in Japan. We stress that it is essential to establish strategies for women with socioeconomic difficulties to encourage ANC, to enroll mothers in ANC earlier, and to improve the quality of programs to control sexually transmitted infections to reduce the frequency of congenital syphilis. These strategies could help prevent cerebral palsy-associated CAM, which may be caused by perinatal infections including syphilis.

## 4. Conclusions

We emphasize that histopathological examination of the placenta should be utilized for investigating the correlation among FHR monitoring patterns and a fetus whose mother was infected with syphilis according to the degree of intrauterine inflammation.
